# Depopulation, immigration, and gender dynamics: a case study of a long-term evaluation

**DOI:** 10.3389/fsoc.2026.1769829

**Published:** 2026-04-30

**Authors:** Leandro Sebastián Fervier, Victoria Sanagustín-Fons, Isabel Pilar Castillo-Salazar

**Affiliations:** 1 Department of Psychology and Sociology, University of Zaragoza, Zaragoza, Spain; 2 Department of Statistical Methods, University of Zaragoza, Zaragoza, Spain

**Keywords:** depopulation, gender dynamics, immigration, repopulation public policy, women in rural areas

## Abstract

This article examines the long-term effects of a local public repopulation policy based on the attraction of international immigrants, implemented in the rural municipality of Aguaviva (Teruel, Spain) since 2000. Using a quantitative, longitudinal, and comparative research design that combines a case study with quasi-counterfactual comparative approach (supported by Synthetic Control Method and Difference-in-Differences), the study analyzes demographic change over a 20-year period (2000–2020) and compares Aguaviva's trajectory with that of 663 rural municipalities in Aragón, grouped by population size and the presence or absence of public intervention. The findings suggest that although the policy was associated with a significant population increase in the medium term, its effects appeared to diminish in the long run. After two decades, Aguaviva's demographic trend shows a tendency to converge with that of municipalities without repopulation policies, reflecting the challenges of immigration-centered repopulation strategies. The analysis also reveals a substantial decline in the foreign population and highlights gender-differentiated dynamics, with women showing a greater tendency to remain in the locality, suggesting unintended policy effects. From the perspective of new institutionalism in sociology, this article emphasizes the importance of longitudinal evaluation and the incorporation of a gender perspective in the design and assessment of public policies aimed at addressing rural depopulation.

## 1 Introduction

Migrations from rural areas to the city have drawn the attention of numerous researchers across different social disciplines (Merino and Prats, 2020). In Spain, the beginning of the rural-to-urban exodus can be traced back to the 19th century, although it increased considerably from the mid-20th century onwards (Alonso-Carrillo et al., 2023; Recaño, 2017). However, it was not until the early 21st century that rural depopulation became a problem on Spain's public and media agenda (Saiz-Echezarreta et al., 2022).

The problem of depopulation is common to propose the settlement of migrants as a potential solution looking for a good life quality standard (Carracedo et al., 2025; Egea and Kreichauf, 2025; Szalanska et al., 2023). In this way, migration and rural depopulation are closely linked in the social imaginary, and the public agenda (Diogo, 2024; Martínez-Carrasco Pleite and Colino Sueiras, 2024). However, historical experiences of mobility and territorial transformation are often overlooked in contemporary debates (García-Madurga et al., 2024; Kvalvaag, 2024).

The first reports on rural depopulation were published in 2000, and in autonomous communities like Aragon, the “*Centro de Estudios sobre Despoblación y Desarrollo de Áreas Rurales*” (CEDDAR) was established (Palacios et al., 2017). Since the emergence of the concept of “Empty Spain” (“*España Vac*í*a”*) (Del Molino, 2016). An example of this is the “*Reto Demográfico,”* which aims to stabilize the rural population and represents a policy response to the problem of depopulation (Rodríguez-Rejas and Díez-Gutiérrez, 2021) although it has also been deemed insufficient (Sáez Pérez, 2021). However, after 20 years of studies and public policies, the results remain discouraging. In addition, there is still a significant lack of analysis of the public policies and programs that have been implemented.

The efforts of authorities and the state, through actors and organizations, fail to stop the exodus that takes place on a daily basis. Despite millions of euros invested in public policies aimed at preventing the migration of citizens from small towns to cities, these efforts are not enough to meet the intended objectives.

While there is a considerable body of research on migratory movements and repopulation processes, most studies approach these dynamics from demographic or migratory perspectives (Camarero, 2020; Camarero et al., 2023; Collantes et al., 2010; García, 2017; Palacios et al., 2017; Pinilla and Sáez, 2017, 2021; Recaño, 2017; del Romero Renau and Pizarro, 2013; Sáez Pérez et al., 2008, 2016; San Martín González and Soler Vaya, 2024). These works mainly describe population dynamics, paying limited attention to the analysis and evaluation of public repopulation policies implemented by local, regional, or national governments. This gap in the literature is precisely what this study aims to address by examining the long-term demographic trajectories associated with such policies in rural areas.

In order to explore this research gap and contribute to the empirical exploration of public repopulation strategies, the following research questions have been developed:

RQ1: What has been the impact of public repopulation policies based on the settlement of international immigrants on the demographic evolution of rural municipalities such as Aguaviva in the long term?

Additionally, the following complementary research questions are proposed:

RQ2: To what extent has Aguaviva's total population changed since the implementation of the repopulation program, compared to similar municipalities where no such policies were applied?

RQ3: What role has international immigration played in the transformation or stabilization of local demographic dynamics?

RQ4: How does sex/gender influence the outcomes and sustainability of repopulation policies based on the settlement of foreign immigrant populations?

Our approach stems from empirical evidence showing that strategies based on attracting foreign populations to depopulated areas, such as in the case studied, often fall short of expectations. This challenges the prevailing view that active state intervention is always necessary to repopulate “Empty Spain.” Public decisions are frequently influenced by normative assumptions, nostalgic imaginaries, or rational planning models that may not align with reality (Sánchez-Oro Sánchez et al., 2020), as well as by cognitive biases that hinder critical evaluation (Kahneman, 2003). In light of this, we argue for a revision of the current paradigm and the adoption of alternative conceptual frameworks to rethink public strategies (Kuhn, 2004).

The general objective of this research is to explore the outcomes and associated demographic shifts of applying a public repopulation policy program based on the attraction of international immigrants. To support this exploration, a Synthetic Control Method (SCM) analysis is conducted to strengthen the robustness of the comparative findings, complemented by a Difference-in-Differences (DiD) model to identify statistical divergences in demographic trends.

The specific objectives are as follows:

i) To analyze the demographic evolution over a 20-year period, considering the implementation of a political repopulation program.

ii) To examine the impact of the immigrant population on local demographic dynamics, and

iii) To observe the influence of sex/gender in the outcomes of repopulation policies based on the settlement of foreign immigrant populations.

The hypothesis is that, in the absence of public repopulation policies, Aguaviva's demographic trajectory would not have differed significantly from that of other rural villages in the Aragon Region, where similar patterns of population decline have persisted over the past 20 years. This challenges the dominant political narrative that seeks to “rescue” rural areas, an impulse often driven by nostalgia or an idealized vision of the past, despite the fact that most people continue to prefer urban life (Niedomysl and Amcoff, 2011).

## 2 Theoretical and conceptual framework

### 2.1 Institutional foundations for the analysis of rural public policy

This research adopts the new institutionalism in sociology (NIS) as its analytical framework, an approach situated within the sociology of public policy. This approach provides an analytical framework for exploring the role of institutions in shaping organizations, which are conceived not as mere functional aggregates, but as social constructions sustained by shared norms, myths, and rules. According to (Meyer and Rowan 1992), organizational structures adopt “rationalized myths” to legitimize their existence, beyond their operational efficiency, while (DiMaggio and Powell 1997) explain structural homogeneity through isomorphic mechanisms (coercive, mimetic, and normative) that operate within organizational fields. (Jepperson 2001) complements this view by arguing that institutions, as historically reproduced social patterns, are not only socially constructed but also regulate action, providing normative and symbolic stability to organizations.

This theoretical framework proves particularly useful for the analysis of public policies, as these are designed, implemented, and evaluated within specific institutional contexts that constrain or expand the range of action available to actors. (March and Olsen 2009) propose a “logic of appropriateness” to explain institutionalized decision-making, in which individuals are guided not solely by rational calculations of consequences, but by internalized norms, routines, and codes of conduct. From this perspective, political behavior is understood through established practices that shape the preferences and courses of action of public actors (de la Rosa Alburquerque, 2019; Vergara, 1993).

Thus, institutions not only stabilize expectations but also provide meaning, predictability, and coherence to public decisions (Jepperson, 2001). Within this framework, actor-centered institutionalism (Olmedo, 2018; Scharpf, 2018) complements NIS by emphasizing how institutional rules condition strategic interactions, allocate resources, and define opportunity structures. This is especially relevant in the analysis of rural or territorial policies, where the persistence of institutionalized routines and historical inertia can account for both the limits to change and the conditions for potential transformation (Zurbriggen, 2006).

This case represents an example of public policies aimed at reversing demographic decline through sustained institutional actions. As noted by (Rayner and Howlett 2009) and (Subirats Humet 2005), policies are both past and future-oriented, insofar as they draw upon lessons from previous experiences to formulate strategies aimed addressing demographic challenges.

To apply the theoretical approach developed to the case study, it is necessary to clarify several fundamental analytical categories. First, defining what is meant by public policy requires conceptual delimitation, given the breadth and heterogeneity of existing approaches since the field's origins with the work of (Lasswell 2013).

This research draws on the definition proposed by (Velásquez Gavilanes 2009), who conceives public policies as processes composed of actions, agreements, decisions, inactions, disagreements, and instruments promoted by public sector actors, potentially with the participation of private actors, aimed at intervening in a situation perceived as problematic. These policies emerge within a specific environment that they seek to modify or preserve, and their analysis requires consideration of institutional elements, as well as the actors involved and their capacities for intervention.

Second, the concept of rural depopulation is defined as the sustained process of demographic decline in small localities, primarily caused by migration from rural to urban areas (Li et al., 2019). This phenomenon, which is both territorial and structural in nature, is reflected in population decreases relative to previous periods, and is driven by the interplay between by negative natural growth (when deaths exceed births) and adverse migratory balances. Depopulation has direct effects on the social, economic, and environmental fabric of communities, leading to the closure of schools, health centers, and basic services, thereby reinforcing the cycle of abandonment and precariousness (Merino and Prats, 2020; Pinilla and Sáez, 2017).

Finally, the concept of a rural municipality is addressed, as it defines the territorial unit under analysis. According to Law 45/2007 on the Sustainable Development of Rural Areas (Jefatura de Estado de España, 2007), a rural municipality is one with fewer than 30,000 inhabitants and a population density below 100 inhabitants per km^2^, with municipalities of fewer than 5,000 inhabitants classified as “small rural.” However, in this study, we adopt the methodological criterion proposed by (San Martín González and Soler Vaya 2024), whereby rural municipalities are defined as those with fewer than 2,000 inhabitants. Moreover, we incorporate the criterion that such municipalities are not part of the Zaragoza Metropolitan Area, in line with (López Jiménez et al. 2002). This delimitation reflects the need to distinguish cases with markedly different demographic and structural dynamics.

### 2.2 Institutional mechanisms and empirical expectations

To strengthen the link between the institutional framework and the empirical analysis, it is necessary to make explicit the mechanisms through which institutions may shape demographic outcomes. This study does not assume that attraction policies generate automatic effects; rather, it posits that such outcomes are mediated by specific institutional configurations operating under a logic of path dependency. As (Pierson 2000) argues, municipalities experiencing prolonged decline develop economic and organizational structures that tend toward self-reinforcement, creating institutional inertia that can hinder attempts to alter pre-existing trajectories through isolated interventions. In rural contexts, this inertia manifests as institutional isomorphism, as conceptualized by (DiMaggio and Powell 1983), whereby labor market structures and closed social networks may constrain local agency, subordinating demographic innovation to broader regional structural trends.

Additionally, a mechanism of selective institutional coupling operates, whereby the effectiveness of repopulation efforts is often contingent on the local capacity to integrate new residents. Following (Stockdale 2006), when policy design focuses exclusively on attraction while neglecting retention through economic and social embedding, the result tends to be a fragile and reversible form of demographic incorporation. This fragility is directly linked to the structural reproduction of the local economy: if the intervention does not substantively transform the economic base, the conditions that expel the most dynamic segments of the population may persist. As (Sampedro Gallego 1996) notes, the lack of occupational diversification disproportionately affects young people and women; therefore, without modifying these structural determinants, policy interventions risk generating only short-term upticks that fail to alter the underlying trajectory of structural decline.

Taken together, these mechanisms allow us to formulate an empirical expectation: if the intervention fails to disrupt these institutional dynamics, the municipality's trajectory is expected to converge with that of comparable territories, a proposition that the subsequent empirical analysis explores in detail.

## 3 Case and methodology

### 3.1 Empirical context and case description

**Figure 1:**
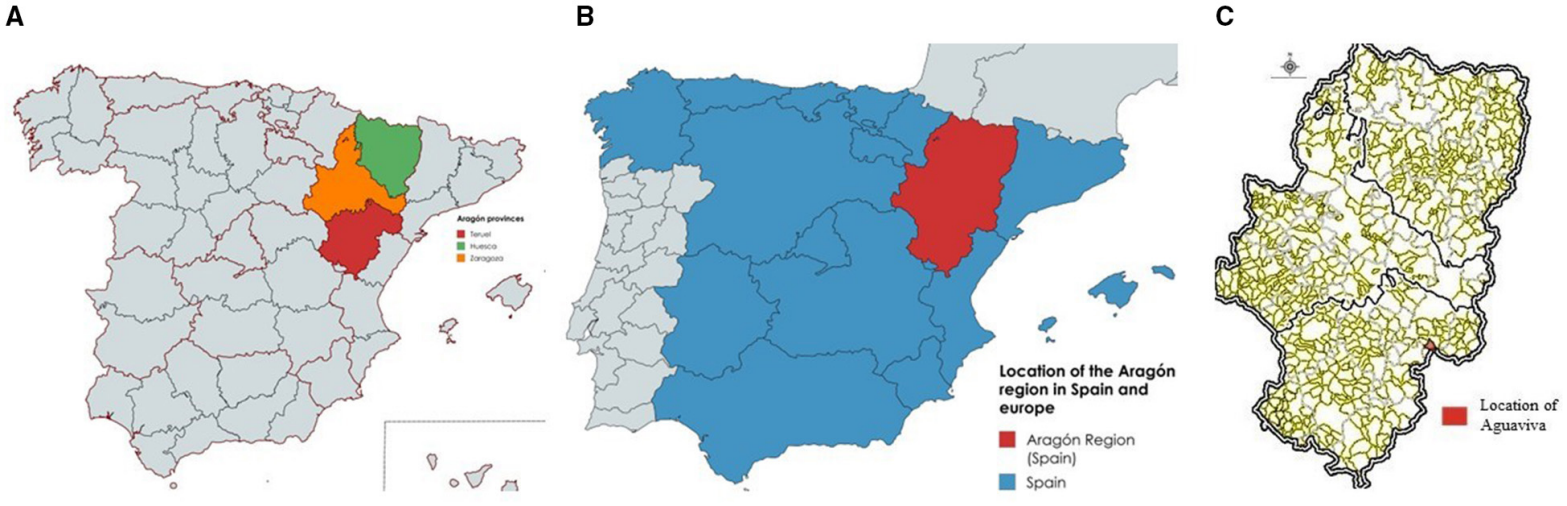
Geographic location of the study area. **(A)** Location of the Aragón region in Spain. **(B)** Provinces of Aragón. **(C)** Location of Aguaviva. Source: Created by the authors using MapChart **(A, B)** and the Aragón Spatial Data Infrastructure **(C)**.

Spain is administratively divided into 17 autonomous communities and 2 autonomous cities, comprising a total of 50 provinces and 8,131 municipalities. One of these communities is Aragón, located in the northeast of the country (see Figure 1A, B and C), which includes the provinces of Zaragoza, Huesca, and Teruel, and contains 731 municipalities in total. Within this region, the province of Teruel stands out as one of the most extreme cases of depopulation in Spain, to the extent that it has been described as a true “demographic desert” due to its population density falling below 10 inhabitants per square kilometer (Ayuda Bosque et al., 2000).

A particularly illustrative example of this phenomenon is the province's population decline over the 20th century: from 251,994 residents in 1900 to just 135,858 in 2001. The municipality of Aguaviva, located within Teruel, clearly reflects this demographic trend, having dropped from 1,781 inhabitants in 1900 to only 592 in 2000.

The case selected for this research is the municipality of Aguaviva, in the province of Teruel. This selection is based on its pioneering role in the design and implementation of local public policies aimed at reversing depopulation, as well as its active engagement in the formation of institutional networks to address rural decline. Notably, Aguaviva played a key role in the creation of the Spanish Association of Municipalities Against Depopulation, a project initiated by its then-mayor (García, 2007; Sáez Pérez et al., 2016).

The municipality's political trajectory is also of analytical relevance. In 1991, Luis Bricio Manzanares assumed office as mayor of Aguaviva, initially representing the People's Party. In 2005, he shifted to the Mixed Group and remained in office until 2013, when he resigned amidst a political scandal. His tenure was instrumental in the implementation of local repopulation strategies and in the articulation of institutional responses to rural depopulation.

In the year 2000, the Aguaviva City Council launched a local repopulation program focused on attracting foreign immigrant populations, specifically Argentine families. This initiative was promoted by the then-mayor, Bricio Manzanares, who personally traveled to Argentina to engage with potential migrants interested in settling in the municipality (Collantes et al., 2010; García, 2017; Sáez Pérez et al., 2016). The program established specific eligibility criteria for the families: having children, not holding a university degree, and committing to reside in Aguaviva for a minimum of 5 years. In return, economic support for relocation and settlement was provided, along with access to adequate housing and job offers within the local labor market (AEMD, 2008).

The immediate impact of the policy was significant: between 2000 and 2003, 55 families relocated to Aguaviva, comprising 112 adults and 144 minors. Beyond its direct scope, the program acquired broader relevance by becoming a reference experience in addressing rural depopulation. In fact, its implementation directly contributed to the establishment of the Spanish Association of Municipalities Against Depopulation, composed of 49 localities in Aragón (33 in Teruel, 12 in Huesca, and 4 in Zaragoza) with populations ranging between 100 and 2,000 inhabitants (AEMD, 2008). This association politically articulated a shared problem and created an institutional platform for promoting coordinated policies in response to the demographic challenge.

From the theoretical framework adopted in this research, the case of Aguaviva offers a privileged opportunity to observe how a local public policy emerges, becomes institutionalized, and reconfigures social dynamics within a rural context marked by depopulation. In terms of the new institutionalism in sociology (NIS), this experience allows for an analysis of how local actors, particularly municipal political leadership, activate mechanisms of institutional change that challenge established routines and norms, fostering new forms of public intervention grounded in the legitimization of innovative practices.

Moreover, the quasi-counterfactual comparative approach enables a detailed exploration of demographic trends by contrasting Aguaviva's demographic and social evolution with alternative non-intervention scenarios reconstructed through the control groups. This approach contributes to a deeper and more contextualized understanding of the phenomenon, while also offering a systematic comparison of local public policies in territories facing demographic crises.

In this study, the term “quasi-counterfactual” is employed in a comparative rather than experimental sense, referring to the systematic contrast between Aguaviva and a set of structurally similar municipalities in the absence of a strictly causal evaluation design.

### 3.2 Methodology

This research adopts a quantitative, longitudinal, descriptive, and comparative approach (Hernández Sampieri et al., 2006), with a retrospective perspective and grounded in a case study (Gerring, 2007; Peña Collazos, 2009; Yin, 2018) combined with a quasi-counterfactual comparative approach (Blasco and Casado, 2009). The analysis is structured around the comparison between Aguaviva and four groups of rural municipalities in Aragón, selected based on population size and the presence or absence of repopulation policies. These groups function as comparative control groups relative to the “treatment” received by Aguaviva.

To ensure statistical comparability, the Synthetic Control Method (SCM) is implemented as the central analytical framework to construct a weighted synthetic “twin” of the municipality, thereby generating a more precise counterfactual trajectory against which the observed demographic evolution can be assessed (Abadie et al., 2010). Additionally, to account for differences in demographic scale across units, the data were subsequently transformed into standardized scores (Z-scores). This standardization allows for the assessment of relative variation and facilitates the application of a Difference-in-Differences (DiD) econometric model as a complementary instrument aimed at identifying statistical divergences from a shared territorial inertia (Angrist and Pischke, 2009). Given that this is a single-case study, the DiD specification is employed as a descriptive and comparative tool to provide suggestive evidence of policy impact rather than as a mechanism for formal causal inference.

The unit of analysis is the municipality, and the universe comprises all 731 municipalities in Aragón as of 2020. From this, an intentional sample of 663 rural municipalities was constructed, defined as those with fewer than 2,000 inhabitants and located outside the metropolitan area of Zaragoza, following the classification by (López Jiménez et al. 2002). This sample represents 90.82% of the territorial units in Aragón and approximately 19.39% of its population in the year 2000. Medium term is defined as a period between 5 and 15 years, whereas long term refers to durations longer than 15 years (Pinilla and Sáez, 2021; Rodríguez, 2003). This empirical design enables a systematic comparison of demographic trends across territorially and structurally similar contexts.

**Figure 2:**
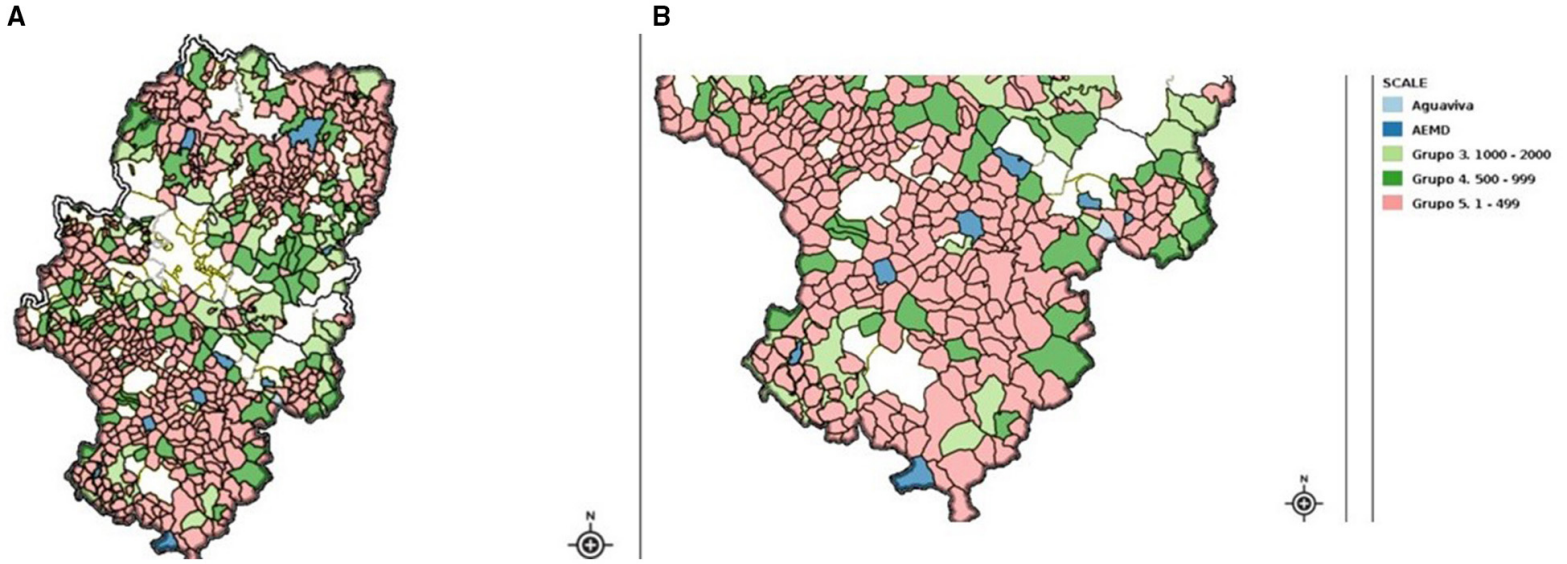
Spatial distribution of Aguaviva and control groups. **(A)** Location within the Aragón region. **(B)** Location within Teruel province. Source: Created by the authors using the Aragón Spatial Data Infrastructure.

The municipalities were classified into five analytical groups: Group 1: Aguaviva (case study); Group 2: Municipalities that were members of the Spanish Association of Municipalities Against Depopulation (AEMD); Group 3: Municipalities with 1,000–2,000 inhabitants and no known intervention; Group 4: Municipalities with 500–999 inhabitants and no intervention; Group 5: Municipalities with 1–499 inhabitants and no intervention (see Figure 2).

This study follows a hypothetico-deductive logic aimed at empirically examining whether the implementation, or absence, of a public repopulation policy is associated with significant long-term demographic differences (González Morales, 2017). The analysis prioritizes the SCM to reconstruct the counterfactual trajectory while the DiD estimator, which compares changes in outcomes over time between the study group (Aguaviva) and the control groups. Epistemologically, it is grounded in the empirical-analytical paradigm typical of applied sociology, combining tools from public policy analysis and evaluation with interpretive frameworks from new institutionalism in sociology (NIS) (Gómez, 2014; Goodman, 1983; Lewis, 1986). This allows for a comparison between observed outcomes and alternative non-intervention scenarios (Artes and Rodríguez-Sánchez, 2022).

The SCM analysis was performed to construct the synthetic counterfactual and conduct the empirical *p*-*value* placebo tests. Complementarily, data processing was conducted using IBM SPSS Statistics v.23, employing General Linear Models (GLM) to estimate the DiD regressions and assess the interaction effects between the “group” factor and the “time” factor (pre- and post-intervention/crisis). To contextualize the case, a purposive sample of 663 municipalities with similar structural characteristics was constructed. The parallel trends assumption was explored through a pre-intervention placebo test. This design strengthens the comparative analysis by ensuring that the observed changes can be analyzed in relation to the policy implementation rather than to pre-existing structural differences. While the SCM and DiD models provide statistical support for the findings, the single-case treatment structure implies that the results should be interpreted as suggestive evidence of policy impact.

Secondary sources were used, specifically from the Spanish National Statistics Institute (INE) and the Aragonese Institute of Statistics (IAEST), covering the period 2000–2020 (see Table 1).

**Table 1:** Variables, dimensions, and indicators.

Variable	Dimension	Indicators
Demographic evolution	Population growth	Total number of inhabitants
		Annual percentage variation
	Residence by nationality	Number of foreign residents
		Foreign population rate
Sex	Sex-based evolution	Male and female population
		Masculinity index

Author's elaboration.

## 4 Results and discussion

### 4.1 Results

Data analysis in the public policy process is a central element. However, in many cases, the results are not analyzed in the long term. This lack of analysis disrupts the virtuous circle of public policies, preventing change when failures are observed (Subirats Humet, 2005).

#### 4.1.1 General demographic evolution (2000–2020)

First, the overall demographic evolution of the municipality during the analyzed period is presented.

**Figure 3:**
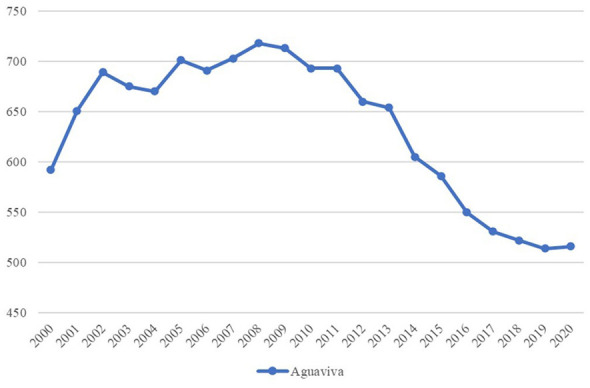
Total population of Aguaviva, 2000–2020. Source: Authors' elaboration based on data from the Spanish National Statistics Institute (INE).

The demographic evolution of Aguaviva was observed from 2000 to 2020. The result between the start and the end of the analyzed period shows a loss of 12.84% of inhabitants (2020 = 516 inhabitants, 2000 = 592 inhabitants (516/592^*^100 = −12.84%) (see Figure 3).

Subsequently, the same procedure was applied to the other groups constructed. Thus, regarding the total population, it can be seen that all the groups have lost residents (see Table 2).

**Table 2:** Population change between 2000–2020.

Group	Category	Population (2000)	Population (2020)	Change (%)
Group 1	Aguaviva	592	516	−12.8
Group 2	AEMD	6,962	6,570	−5.63
Group 3	2,000–1,000	68,226	64,062	−6.1
Group 4	999–500	58,952	49,439	−16.1
Gruop 5	499–1	96,043	80,167	−16.5

Authors' elaboration based on data from the Spanish National Statistics Institute (INE).

Comparing the population evolution of Aguaviva with the other constructed groups, it can be seen that the loss of residents in the case of Aguaviva and in Group 4 (500 to 999 inhabitants) are similar (16.1% for Group 4 and 12.8% for Aguaviva).

**Figure 4:**
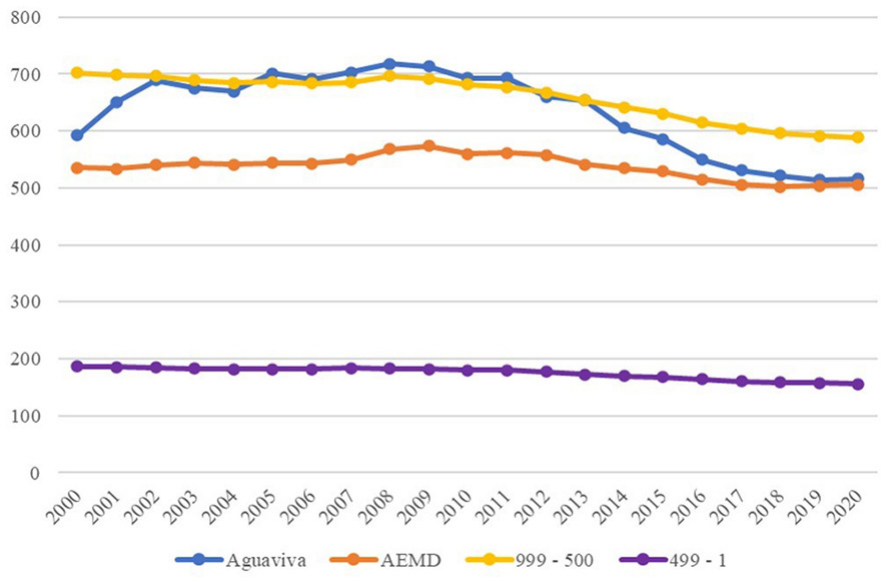
Average population evolution, 2000–2020. Source: Authors' elaboration based on data from the Spanish National Statistics Institute (INE).

In Figure 4, the average population of each group was calculated, and Group 3 was omitted from the graph in order to observe in more detail the evolution of Aguaviva and the rest of the smaller population groups.

When analyzing the evolution over time, Figure 4 shows that Aguaviva diverges from Group 4, the most similar in terms of population, but then returns to the same dynamics, suggesting that in the medium term (10 years) public policy appears to have had a positive influence, but fails to maintain these results in the long term (20 years).

**Figure 5:**
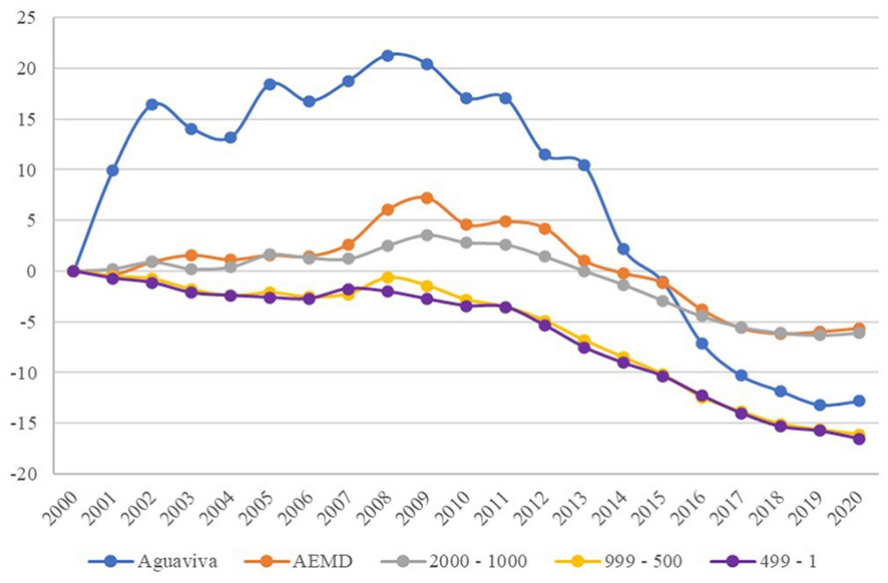
Population variation (%) between 2000–2020. Source: Authors' elaboration based on data from the Spanish National Statistics Institute (INE).

The percentage variation of the population year by year in relation to the year 2000 was also analyzed. Figure 5 illustrates a notable divergence in the medium term, followed by an abrupt decline in the long term, ultimately ending in a similar situation to that of other groups included in this research.

#### 4.1.2 Migration dynamics and composition by origin

Second, the evolution of the foreign population and the relationship between the native and foreign populations are analyzed. According to the data available from the (INE 2024), Aguaviva had 592 inhabitants in 2000. Unfortunately, statistics broken down by nationality are not available until 2003. However, 3 years after the implementation of the public repopulation policy in Aguaviva, the number of inhabitants had increased to 675 people, of which 556 were Spanish and 119 were foreigners. By 2020, the number of foreigners had decreased to 63, representing a drop of 47.06%, according to INE data (2024). Meanwhile, Spaniards dropped from 556 residents in 2003 to 453 in 2020, reflecting an 18.6% decrease in population over the analyzed period.

**Figure 6:**
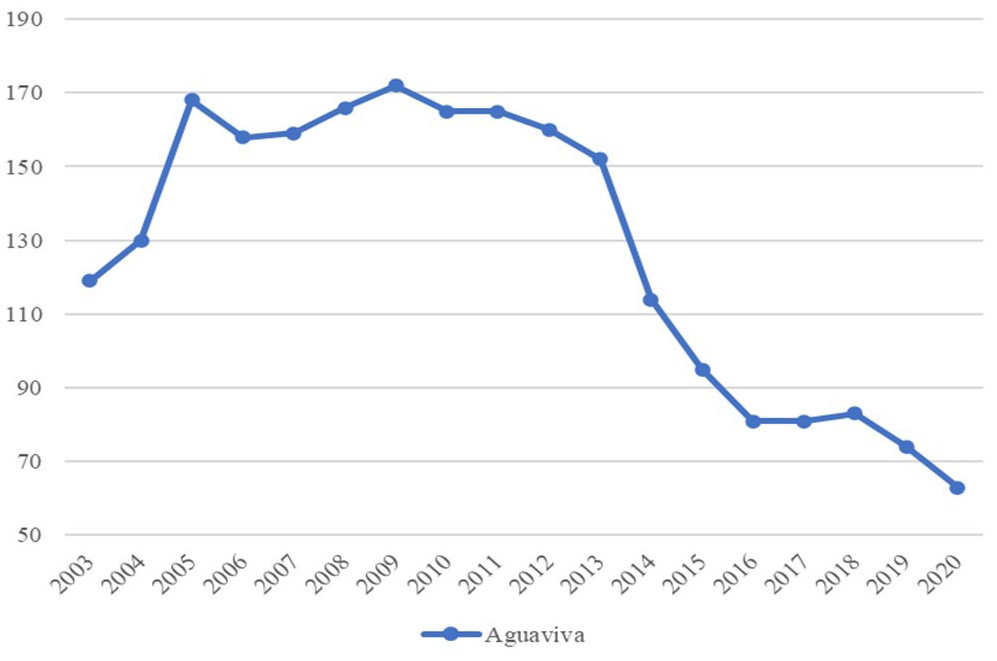
Foreign population of Aguaviva 2003–2020. Source: Authors' elaboration based on data from the Spanish National Statistics Institute (INE).

The evolution of foreign residents in Aguaviva shows a rapid upturn until 2005 and then a slight increase until 2009. After that, a slow decline starts until 2013, with a sharp acceleration in the loss of inhabitants until 2016, and then again, a gradual decrease until 2020 (see Figure 6).

Table 3 shows the variation in the foreign population between 2003 and 2020, showing disparate behavior. While in Aguaviva the foreign population decreases considerably, in the other groups the population increases significantly.

**Table 3:** Foreign population 2003–2020.

Group	Category	2003	2020	Change (%)
Group 1	Aguaviva	119	63	−47.06
Group 2	AEMD	368	830	125.54
Group 3	2,000–1,000	3,291	8,779	166.76
Group 4	999–500	1,943	4,954	154.97
Group 5	499–1	2,635	6,714	154.80

Authors' elaboration based on data from the Spanish National Statistics Institute (INE).

**Figure 7:**
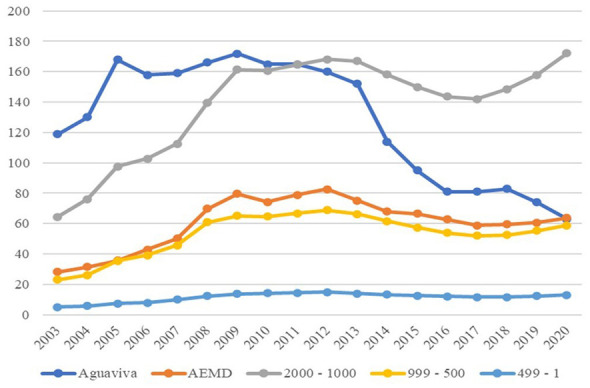
Foreign population (average) 2003–2020. Source: Authors' elaboration based on data from the Spanish National Statistics Institute (INE).

All the analyzed groups have seen an increase of more than 100% in the number of foreign residents between 2003 and 2020, as shown in Table 3. Meanwhile, Aguaviva has experienced a 47.06% loss of foreign residents over the same period of time. For this reason, the data reflects a trend consistent with an unintended trajectory of the policy, as more foreigners settle in a locality, over the same period of time, the more people emigrate, even surpassing the levels seen at the beginning of the immigrant attraction process, as shown in Figure 7.

**Figure 8:**
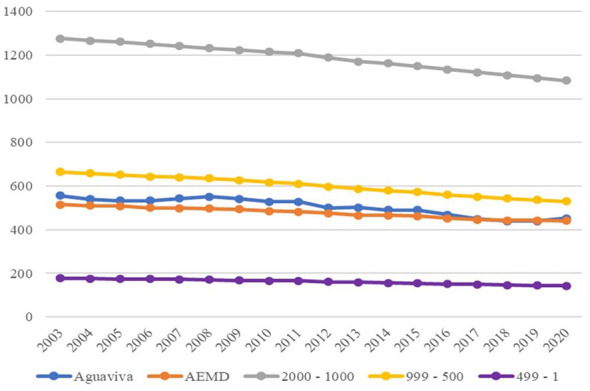
Spanish population (average) 2000–2020. Source: Authors' elaboration based on data from the Spanish National Statistics Institute (INE).

Figure 8 does not show abrupt variations in the Spanish resident population compared to the foreign one, showing a rather slight and steady decline over the period.

**Figure 9:**
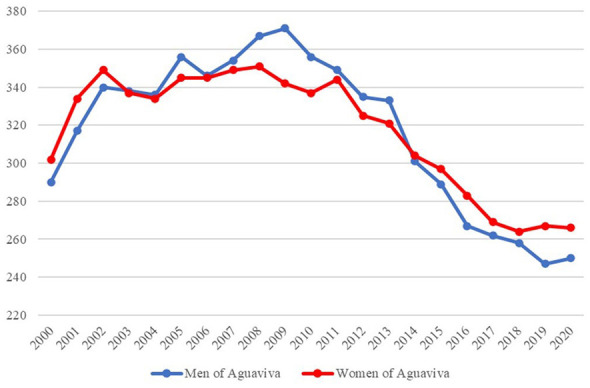
Population by sex 2000–2020. Source: Authors' elaboration based on data from the Spanish National Statistics Institute (INE).

When analyzing the population by sex, a trend can be observed in which the female population is larger during the first stage of the public repopulation policy (2000–2002). Then, the male population increases, but they are also the ones who emigrate the most from Aguaviva. Likewise, the curves show similar trends, suggesting that immigrants have settled with their “traditional” families and that, eventually, when they leave Aguaviva, they do so with their families as well (see Figure 9).

Although the influx of foreign population introduces variations in the demographic structure, no sustained transformation of the overall trend is observed.

#### 4.1.3 Gender dimension

**Figure 10:**
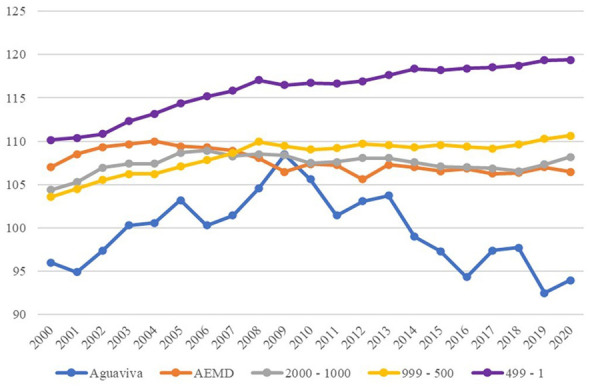
Masculinity index 2000–2020. Source: Authors' elaboration based on data from the Spanish National Statistics Institute (INE).

Third, the evolution of the masculinity index and the sex distribution is examined. When the masculinity index (number of men per 100 women) is constructed for each group and compared with Aguaviva, it suggests that the public repopulation policy may have an effect on gender distribution. While the masculinity index tends to increase in all groups, in Aguaviva it decreases. This suggests that the policy trajectory in Aguaviva is associated with a potential rooting effect among women, a situation that was not anticipated in the policy design (see Figure 10).

The incorporation of a gender perspective refines the policy evaluation, showing that the observed changes do not structurally alter the underlying demographic imbalances.

#### 4.1.4 Synthetic control method (SCM) analysis

To explore and provide statistical support for the observed trends, a counterfactual was estimated using the Synthetic Control Method (SCM). This approach constructs a synthetic unit that reproduces Aguaviva's pre-intervention trajectory through a weighted combination of comparable rural municipalities. The donor pool consisted of 649 rural municipalities, excluding the treated unit and those municipalities exposed to similar repopulation policies, thereby preventing contamination of the counterfactual.

Optimization was conducted jointly for total, male, and female population, imposing a single weight vector (W). This strategy ensures structural demographic coherence (Total = Men + Women) and avoids the generation of mutually inconsistent synthetic units. The optimization was performed over the available pre-intervention period (1996, 1998, and 1999), excluding 1997 due to missing data. The pre-treatment fit was extremely high, with a pre-treatment RMSPE of 0.000092 for total population, indicating an almost exact replication of the baseline trajectory without reaching artificially null values.

**Figure 11:**
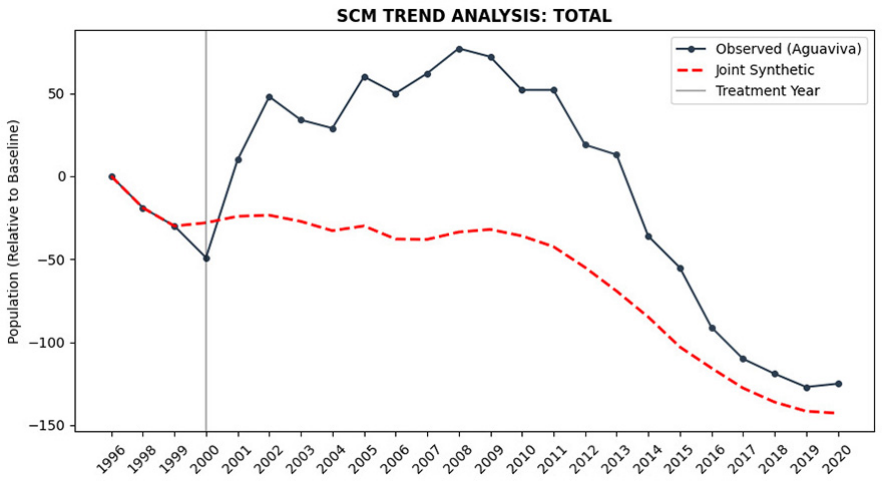
SCM trend analysis: total population. Source: Authors' elaboration using INE data and Python. Observed trajectory for Aguaviva vs. synthetic control (1996 baseline). Vertical line indicates policy onset (2000).

Figure 11 presents the observed trajectory relative to the synthetic control. During the first phase of the policy (2000–2008), Aguaviva exhibits a marked positive divergence from its counterfactual, with an average ATE of +66.24 inhabitants. This result suggests a substantively meaningful initial impact associated with the intervention. In the second period (2009–2020), the differential partially narrows but remains positive, with an average ATE of +52.56 inhabitants. The trajectory displays a process of gradual convergence, indicating that the initial advantage tends to attenuate over time.

The joint analysis further reveals that during 2009–2020 the female-specific effect reaches +24.69 inhabitants, maintaining additive consistency with the total effect. Although non-parametric inference does not reach conventional levels of statistical significance, the magnitude and persistence of the female differential are consistent with the results of the Difference-in-Differences (DiD) analysis presented in the following section regarding the reduction of the masculinity index. Substantively, the SCM is consistent with a relative modification in demographic composition compared to structurally comparable municipalities.

**Figure 12:**
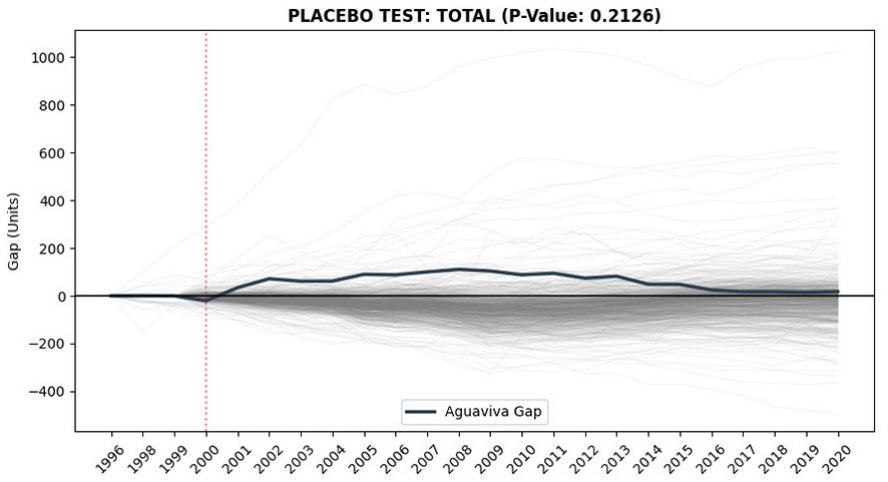
In-space Placebo Test (Total population). Source: Authors' elaboration using INE data and Python. Gap distribution (Observed-Synthetic) for Aguaviva (dark blue) vs. 649 placebo units (gray). Empirical *p*-value: 0.2126.

Statistical validity was assessed through in-space placebo tests applied to the 649 municipalities in the donor pool (Figure 12). The empirical *p-value* based on the Post/Pre RMSPE ratio distribution was 0.2126 for total population, placing Aguaviva in the upper segment of the distribution, albeit without achieving conventional statistical significance. Given the limited number of pre-treatment years (1996–1999), inference should be interpreted cautiously. In this context, SCM is primarily employed as a structural robustness check. This evidence is further complemented by the econometric estimation of a DiD model in Section 4.1.5, which allows for a formal testing of the intervention's statistical significance.

A crucial observation from Figure 11 is the trajectory of Aguaviva after 2016. While the municipality showed a notable divergence during the first decade of the policy, this advantage is almost entirely erased in the final years of the series. The eventual convergence between Aguaviva and its synthetic counterpart suggests that the policy's impacts were temporary and failed to induce a permanent structural change. This “fade-out” effect indicates that the initial demographic boost was not self-sustaining, leading to a return to the inertial dynamics of the donor pool.

**Figure 13:**
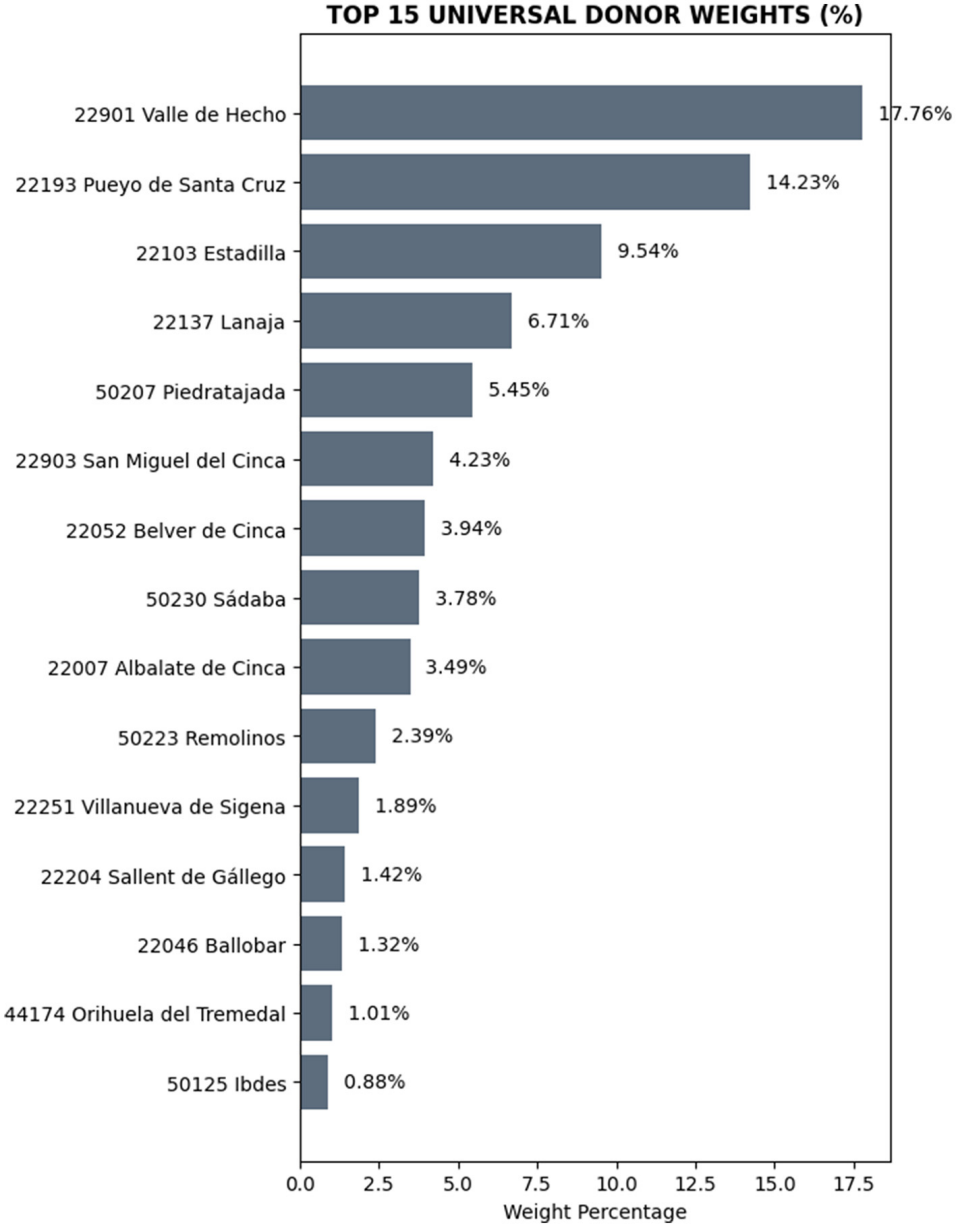
Composition of the synthetic counterfactual. Source: Authors' elaboration using Python. Percentage weights (*W*) of the leading donor pool municipalities forming Aguaviva's synthetic unit.

Aguaviva's synthetic “twin” was constructed as a weighted combination of municipalities from the donor pool (Figure 13). This donor selection ensures that the counterfactual does not rely on a single municipality but instead on an optimized weighted average capturing the structural characteristics of rural Aragon.

#### 4.1.5 Econometric analysis: difference-in-differences (DiD) as complementary evidence

To provide further statistical support for the observed trends, and to test the significance of the divergence identified in the previous section, a Difference-in-Differences (DiD) model was estimated using standardized scores (Z-scores). This approach aims to identify the statistical divergence of the repopulation policy from the inertial dynamics of the control municipalities, under the parallel trends assumption in the pre-intervention period.

Prior to estimating the observed variation, a placebo test was conducted to verify the internal consistency of the model. An intervention was simulated for the period 2000–2003 (pre-policy phase) in order to assess whether Aguaviva already exhibited a divergent trajectory due to exogenous or incidental factors. The results revealed no statistically significant differences between Aguaviva and the control groups (*p* = 0.812), providing support for the parallel trends assumption. This finding is relevant, as it suggests that the subsequent demographic divergence is consistent with the timing of the policy intervention rather than pre-existing trends.

The analysis shows that the intervention in Aguaviva was associated with a statistically significant difference in its relative temporal evolution compared to the other groups. The between-subjects effects model indicates a significant interaction between the group and time factors (Treatment × Year), with *p* = 0.044 (see Table 4). This result is important, as it indicates that Aguaviva's trajectory diverged from regional trends in a statistically significant way following policy implementation.

**Table 4:** Results of the Difference-in-Differences (DiD) model for total population (Z-scores).

Source of variation	*df*	Mean square	*F*	Sig. (*p-value*)
Corrected model	3	2.841	18.45	0.000
Group factor (Aguaviva vs. Controls)	1	1.120	7.27	0.008
Time factor (Pre vs. Post Policy)	1	6.540	42.46	0.000
DiD interaction (Group × Time)	1	0.634	4.12	0.044
Error	96	0.154		
Corrected total	99			

R^2^ = 0.51. The interaction term (Group × Time) represents the net effect of the repopulation policy. Significance levels (p-values), F-statistics, and coefficients of determination were obtained using the General Linear Model module in IBM SPSS Statistics, ensuring the robustness of the causal inference and control of Type I error.

Source: Author's own elaboration based on data from Instituto Nacional de Estadística (INE), processed in IBM SPSS Statistics v.23.

The overall model fit indicates moderate-to-high explanatory power (*R*^2^ = 0.51), although the substantive interpretation rests primarily on the magnitude and statistical significance of the interaction term.

When disaggregating the data by nationality, the results show that the observed change fell almost exclusively on the foreign population, while the Spanish-national population maintained a persistent decline across all groups analyzed.

The Spanish population exhibits an identical linear decline to that observed in the control groups (*B* = −0.120), signifying that the policy did not significantly alter the out-migration trend of local human capital. By contrast, the foreign population initially drove the structural break but suggest highly vulnerable. From 2009 onward, coinciding with the economic crisis, the slope of decline in Aguaviva (*B* = −0.210) was significantly steeper than in the control municipalities (*B* = −0.085), suggesting that the initial success in attracting immigrant families did not translate into structural embeddedness capable of withstanding macroeconomic shocks (see Table 5).

**Table 5:** Results of the Difference-in-Differences (DiD) model: Foreign vs. Spanish population (2003–2020).

Independent variable	Model 1: Foreign population	Model 2: Spanish population
Aguaviva effect (Treatment)	102.819^***^	−102.989
	(−21.268)	(−155.857)
Crisis effect (Post-2008)	27.984^*^	−58.434
	(−11.649)	(−85.366)
DiD interaction (Differential effect)	−60.901^*^	1.434
	(−26.048)	(−190.885)
Significance (*p-value*)	0.022	0.994
R-squared	0.745	0.18

(1) Standard errors in parentheses. (2) Significance levels: ^***^p < 0.01; ^**^p < 0.05; ^*^p < 0.1. (3) Model 1 explains 74.5% of the variance, whereas Model 2 indicates that the policy does not account for the behavior of the native population.

Source: Author's own elaboration based on data from Instituto Nacional de Estadística (INE), processed in IBM SPSS Statistics v.23.

One of the most relevant findings of the DiD model concerns changes in the Masculinity Index (MI). While the control groups maintain a highly masculinized and temporally stable structure (*p* = 0.897), Aguaviva exhibits a statistically significant divergence (*p* = 0.047; see Table 6).

**Table 6:** Results of the DiD model for the masculinity index (MI).

Source of variation	*df*	Mean square	*F*	Sig. (*p-value*)
Modelo corregido	3	1.452	3.12	0.030
Group factor (Aguaviva vs. Controls)	1	0.980	2.11	0.149
Time factor (Pre vs. Post policy)	1	0.007	0.02	0.897
DiD interaction (Group × Time)	1	2.050	4.41	0.044
Error	96	0.465		
Corrected total	99			

The statistical significance of the interaction term (p = 0.044) suggests that the family-attraction policy altered the gender composition in a manner divergent from the surrounding rural context. Estimates were obtained through ANOVA-based impact models in IBM SPSS Statistics.

Source: Author's own elaboration based on data from Instituto Nacional de Estadística (INE), processed in IBM SPSS Statistics v.23.

This result reinforces the descriptive evidence of a process of relative feminization compared to the surrounding rural environment. The analysis indicates that the policy aimed at attracting family units not only increased population levels but also was associated with the retention of women, thereby reducing the gender gap characteristic of inertial depopulation. The statistically significant temporal interaction (*p* = 0.044) indicates that this increased female presence is not a static condition, but a sustained and progressive deviation from the trajectory observed in the control groups, where masculinization tends to become entrenched over time. Given the single-case treatment structure, these DiD results are presented as suggestive evidence of policy impact rather than definitive proof of causation.

### 4.2 Discussion

#### 4.2.1 Theoretical implications

The findings suggest that the intervention in Aguaviva failed to subvert path-dependent mechanisms or the structural dynamics of the local market. The evidence suggests that path dependency operated as a self-reinforcing process in which, despite the attraction policy, the inertia of a narrow labor market and a service provision structure adapted to decline ultimately prevailed. As (Mahoney and Thelen 2010) argue, local institutions are often subject to strong path-dependent constraints. Such structural inertia generates resistance that isolated interventions rarely manage to dismantle permanently, which explains why, following the initial boost of the migration policy, the municipality ultimately converged with the contractionary demographic trends of its regional environment, as observed in the longitudinal comparison with its structural counterparts.

Moreover, the evidence points to the operation of selective institutional coupling, which appears to have limited the long-term effectiveness of the policy. By prioritizing external attraction over retention and deep integration, the institutional design failed to achieve the “institutionalization of change” necessary to sustain growth. Following (Lowndes and Roberts 2013), without robust anchoring in local rules and community structures, demographic incorporation remains fragile, allowing pre-existing organizational logics to absorb and neutralize the effects of policy innovation.

Finally, the persistence of structural reproduction mechanisms within the local labor market accounts for the continued out-migration of younger cohorts. Because the underlying economic base was not transformed, the municipality appeared unable to retain the demographic surplus initially generated. The absence of sustained divergence from structurally similar rural municipalities suggests that, although the policy produced visible short-term effects, the fundamental drivers of decline remained intact (Li et al., 2019). Ultimately, the pre-existing trajectory reasserted itself, a finding consistent with the eventual convergence between the study case and the comparative control groups. This supports the idea that the sustainability of repopulation depends on the capacity of policy not merely to mitigate decline, but to alter the underlying structural rules of the game. In line with the methodological constraints of a single-case study, these theoretical implications should be viewed as suggestive evidence of the challenges in breaking regional demographic inertia.

#### 4.2.2 Public policy implications

These findings do not imply that attraction-based policies are ineffective in all contexts. Rather, in the case examined, a design primarily centered on attraction proved insufficient to modify long-term institutional dynamics.

In the medium term, the public repopulation policy analyzed resulted in a 21.28% increase in the population of Aguaviva, raising the number of inhabitants from 592 to 718 between 2000 and 2008. By 2008, the town's resident composition was 552 Spaniards and 166 foreigners, reaching a peak of 172 foreign inhabitants in 2009 (INE, 2024). After a decade, the results allow for multiple interpretations. According to (Collantes et al. 2010) the outcome has been positive in demographic terms for the municipality of Aguaviva, but it has lacked positive impacts in other aspects. In the same vein, (García 2017) points out the issues that arose in Aguaviva regarding the cultural and labor integration of the immigrants welcomed.

While the case highlights the challenges of integrating international immigrants in Aguaviva, this should not be generalized, as local dynamics are embedded in complex and context-specific configurations, as demonstrated by (Sætermo and Kristensen 2024) in relation to immigrants in Norway. Moreover, the integration of immigrants into local societies is often more complex in rural than in urban contexts (Zahl-Thanem and Haugen, 2019). Likewise, other authors, such as (Gil-Alonso et al. 2023) analyse the complex relationship between migration and rural development. Their findings on how migration patterns affect the sustainability of rural communities complement our analysis of the Aguaviva case, especially with regard to the failure to retain the immigrant population in the long term.

The temporal dimension of Aguaviva's repopulation policy reveals a complex interplay of factors that initially supported population growth but ultimately failed to sustain it long-term. This pattern of medium-term success (2000–2008) followed by long-term regression (2008–2020) can be attributed to several interconnected factors. First, the initial influx of immigrants benefited from strong institutional support, media attention, and financial incentives that created an immediate demographic impact (Bayona-i-Carrasco and Gil-Alonso, 2013). However, these formal support structures were not matched by sustainable integration mechanisms addressing the sociocultural dimensions of settlement. As (Camarero and Sampedro 2019) argue, successful rural immigration requires not just demographic planning but also sustained investment in social infrastructure that fosters belonging and intercultural dialogue.

Second, the economic trajectory of rural areas played a decisive role in the policy's effectiveness. The initial growth period (2000–2008) coincided with Spain's economic boom, which provided employment opportunities even in rural areas. When the global financial crisis struck in 2008, these opportunities diminished significantly, triggering an outflow of the recently settled immigrant population that had not yet developed deep territorial roots (Pinilla and Sáez, 2021). This economic vulnerability is explicitly reflected in the long-term SCM results (Figure 11). The convergence of Aguaviva's trajectory with its synthetic counterfactual after 2016 suggests that the policy's impacts were essentially temporary. As the initial institutional momentum and favorable economic conditions faded, the demographic “premium” was gradually erased, indicating that the intervention failed to alter the municipality's long-term structural equilibrium. This pattern of convergence suggests that without a profound transformation, attraction-based policies may only offer a transient departure from inertial decline. As (Rye 2018) observes in similar contexts across Europe, economic precarity often leads to sequential migration patterns among foreign workers in rural areas, with small towns serving as temporary settlements rather than permanent homes.

Third, the data points toward a failure of generational succession planning within the repopulation strategy. While the policy focused on attracting families with children, it did not adequately address the future aspirations of these children once they reached adulthood. Research by (Stockdale et al. 2018) indicates that second-generation immigrants in rural areas often face educational and career aspirations that cannot be fulfilled locally, creating a secondary wave of outmigration approximately 10–15 years after initial settlement, precisely the pattern observed in Aguaviva. This generational dimension has been largely overlooked in Spanish repopulation policies but appears crucial to explaining the long-term demographic trajectory revealed by our data.

For the period 2008–2020, Aguaviva lost 28.1% of its population, decreasing the number of inhabitants to 516, of which 440 were Spaniards and only 63 were foreigners. This population decline means that, after 20 years, the population growth had disappeared, resulting in a 12.9% decrease over the period from 2000 to 2020. The research reveals, albeit implicitly, a phenomenon that (Camarero 2020) has conceptualized as the “rural divide” and the consequent social malaise underlying depopulation processes. This dimension is reflected in our results, which show a significant rural exodus process.

Following Lichter's (2012) argument, who warns that immigration processes in rural contexts can reshape demographic and social dynamics, often not without tensions, it is worth asking whether the emigration of Spanish residents from Aguaviva could be, at least in part, linked to displacement processes resulting from the arrival of immigrant populations. Although there is no conclusive evidence to establish a direct causal relationship, perceptions of competition over limited resources, such as employment, housing, or certain services, may have influenced decisions to leave. The findings suggest that the demographic evolution of Aguaviva was not merely a reflection of regional trends but appears to be associated with a response to the implemented policy particularly during the initial policy phase (2000–2008). The internal consistency of these findings does not rely solely on the observation of the data, but on the statistical patterns identified through the model. This divergence is further aligned with results from the Synthetic Control Method (SCM), which indicates that Aguaviva's trajectory surpassed its “synthetic twin” during the initial policy phase, although this specific estimation does not reach conventional significance levels. The observed patterns were further explored through a placebo test applied to the pre-intervention period (1996–1999), which showed no evidence of significant selection biases prior to the analyzed policy milestone. As no significant deviation was detected before policy implementation, the parallel trends assumption receives statistical support, allowing the observed demographic changes to be interpreted as consistent with the timing of the policy rather than to pre-existing dynamics. Similar dynamics have been documented in other rural contexts in Europe and North America, where population recovery has largely relied on international migration flows.

When comparing the case of Aguaviva with the rest of Spain, it can be observed that between 2001 and 2018, 5,120 Spanish municipalities lost population. This means that 63.2% of them have experienced negative population growth. Moreover, it can be noted that 48.3% of Spanish municipalities lost between 10% and 50% of their inhabitants (Ministerio de Política Territorial y Función Pública, 2018), similar to the trends observed in Aguaviva and in the other groups of municipalities constructed in this study.

The public policy analyzed in the case of Aguaviva is particularly striking in relation to the foreigners who had settled in the municipality. Unlike a purely descriptive analysis, the application of the Difference-in-Differences (DiD) model enables an exploration of the policy's potential impact on demographic structure. This distinction, supported by the econometric results, suggests that the policy achieved a selective effect in altering the population trajectory relative to the trends observed in the control groups. The foreign population also declined during this period, dropping by 47.06%, more than twice the decrease seen in the native population. The case analyzed does not align with the findings of (Diogo 2024) who claims that foreigners tend to settle in small towns because they find more opportunities. The Aguaviva case shows that foreigners also migrate away from rural villages after a period of time. Thus, the data from Aguaviva are consistent with the approach of (Recaño 2017) and (Sampedro and Camarero 2018) who show that the settlement of foreigners is a temporary palliative as they eventually migrate again from small rural towns to larger urban centers. The results of this research also align with the findings of (Sáez Pérez et al. 2008) in relation to the design of public policy, suggesting that without citizen participation, such policies are likely to fail in terms of long-term results. In the same vein, (Konduri and Lee 2023) present a successful case in South Korea, where social and citizen participation attracts new residents to localities that facing demographic problems. Similarly, (Veréb et al. 2024) highlight the role of community engagement in promoting sustainable wellbeing in rural communities.

When population dynamics are analyzed by sex, in the case of Aguaviva, there is a trend toward a greater number of men during the population expansion period (2000–2009), although women are in the majority in the first 3 years, followed by a more abrupt decrease in the number of men compared to women (2010–2020). This situation allows us to suggest that men are the first to migrate, followed by women. At the same time, men are more likely to migrate than women when moving from small rural localities. This tendency suggests that it is central to adopt a gender perspective when designing public repopulation policies. (Cruz Souza et al. 2023) highlight the importance of considering gender dynamics in rural development strategies. Their observations on the differential patterns of rootedness between men and women in rural environments provide a theoretical framework that helps explain the results observed in Aguaviva, where women tend to stay more in rural areas.

The observed trend could be interpreted in light of the higher tendency among rural women to form families at an early age, which may reduce their likelihood of migrating again (Brooks and Clark, 2024). Similarly, a difference has been observed between Aguaviva and the other groups. In Aguaviva the masculinity index decreases over time, while in the other groups it tends to increase. This situation contradicts (Recaño 2017), who argues that women are more likely to emigrate, resulting in gender imbalances, as well as (Camarero et al. 2013), who identify the masculinization of rural areas as a consequence of international migration. This behavior may be due to the public repopulation policy implemented in the locality. While the SCM indicates that this comparative advantage in female retention tends to stabilize toward the end of the period, the overall shift remains a distinctive outcome of the intervention compared to the inertial masculinization of the control groups.

As (Brasier et al. 2014) argue, women's multiple identities entail considerable complexity, as they can, and indeed do, fulfill multiple roles within the household, society, and the labor market. However, they remain excluded from key decision-making spaces due to gender norms that limit their access to education, digital skills, and leadership opportunities, while they continue to bear a disproportionate burden of reproductive labor (Huot et al., 2023). Nevertheless, our findings suggest that their role is central to public repopulation policies. Enhancing women's empowerment and gender equity can benefit both women and their households, but if the structural causes of their inequality are not addressed, such policies risk placing the responsibility for family and community wellbeing disproportionately on them (Bain et al., 2020).

Undoubtedly, the long-term evaluation reveals a sense of frustration with the public repopulation policy in Aguaviva through the recruitment of foreign immigrants. Among the causes of the public policy failure in the case analyzed, it is possible to link it to three events. First, the media announced the public repopulation policy in large cities, when they should have focused on attracting people from small villages. Second, the psychological misunderstanding between locals and immigrants who arrive in large numbers, leads to undesirable situations that undermine local cohesion and integration (del Romero Renau and Pizarro, 2013). Finally, immigrants' expectations are not fulfilled once they have settled in the territory, which prevents their assimilation and permanence in the locality (García, 2017).

Moreover, local knowledge about both positive and negative rural dynamics must be taken into account, as it is essential for sustainable rural development and territorial resilience. Newcomers are often sensitive to changes that contradict their expectations, while long-term residents may choose to leave if they perceive an unbridgeable gap between what is, what they imagined, and what lies ahead (Kajdanek et al., 2022). Therefore, the evaluation of intervention programs targeting depopulation is essential to mitigating unintended consequences (Jaraíz-Arroyo et al., 2025).

The SCM results are directionally consistent with the DiD estimates, suggesting a structural pattern in which Aguaviva is positioned in the upper segment of the donor pool distribution, although without reaching conventional levels of statistical significance (*p* = 0.2126). In this sense, while the model's coefficient of determination (*R*^2^ = 0.51) indicates moderate-to-high explanatory power, it is the statistical significance of the DiD interaction term that provides suggestive evidence of a divergence from the trajectory observed in the control group. The quasi-counterfactual comparative approach applied in this research broadens the framework for understanding the phenomenon (Gómez, 2014; Goodman, 1983; Lewis, 1986; Maldonado, 2005, 2022) enabling us to analyse rural depopulation in Spain and one of its possible solutions. This research highlights the ethical need to evaluate rural repopulation policies, especially when they involve significant public spending and directly impact people's lives. The lack of long-term assessments is not only a methodological gap but also an ethical issue, as it commits migrants to promises that may not be fulfilled. It thus emphasizes the responsibility to design evidence-based policies with proper monitoring and accountability mechanisms (Schmitter, 2004). However, given the single-case nature of the study, these SCM and DiD results are presented as suggestive evidence of policy impact rather than definitive causal proof.

#### 4.2.3 Study limitations

Despite the analytical contributions derived from both the Synthetic Control Method (SCM) and the DiD model employed, this study presents certain limitations that should be acknowledged. First, the use of secondary data from the INE restricts the analysis of the foreign population to 2003 onward, limiting detailed observation of the policy's initial 3 years. Second, although the control groups help to contextualize the findings within regional trends, the “single-case” nature of Aguaviva means that the results should be interpreted as suggestive rather than definitive evidence of causality.

In order to partially mitigate this structural constraints, the Synthetic Control Method was implemented, enabling the construction of an optimized counterfactual and reducing the bias inherent in comparing a single case to broad aggregate averages. Nevertheless, caution is warranted when generalizing the results to other regions with different economic structures. In this sense, the statistical results are presented as complementary evidence that supports the descriptive findings, rather than absolute proof of policy causation. Finally, while the model controls for fixed temporal factors, qualitative variables such as social cohesion and local integration are not fully captured by demographic indicators and may influence long-term retention outcomes.

## 5 Conclusions

This research suggests that public repopulation policies must be evaluated in the long term, rather than being assessed solely based on their short and medium-term impacts. The policy implemented in Aguaviva appears to have faced significant challenges in achieving a sustainable long-term impact, despite showing promising results in the medium term. While the Synthetic Control Method (SCM), which constitutes the main counterfactual estimation strategy, does not show a statistically significant effect (*p* = 0.2126), the complementary Difference-in-Differences (DiD) analysis indicated a statistically significant divergence during the implementation phase (*p* = 0.044), a pattern that finds descriptive parallels in the Synthetic Control Method, the locality was ultimately unable to maintain its population; on the contrary, after 20 years, it continued to lose inhabitants at a rate similar to that of other municipalities in Aragón. This highlights the need to incorporate longitudinal evaluation mechanisms to assess the potential effectiveness of implemented policies.

Moreover, the research indicates that attracting foreign populations to rural villages does not automatically ensure demographic sustainability. Although the arrival of immigrants initially appeared to reverse the local population decline, the long-term impact showed extreme vulnerability to external shocks like the 2008 crisis. This situation underscores the fact that retaining population in small rural towns requires multiple and sustained incentives beyond initial ones and a focus on structural integration rather than mere attraction.

The incorporation of a gender strategy in public repopulation policies is presented here as a strategically relevant factor. This conclusion is based on two key findings that may also serve as starting points for future research. First, women tend to settle more readily during the initial phase when repopulation opportunities emerge. Second, they are observed to remain in their new place of residence counteracting the inertia of masculinization typical of rural decline. While robustness checks suggest that this comparative advantage tends to converge with regional trends in the long run, the observed trends during the growth phase point to the potential value of a gender-sensitive approach.

In relation to the initial hypothesis, the quasi-counterfactual comparative approach conducted in this research suggests that repopulation policies in Aguaviva have shown low efficiency due to factors consistent with path dependency and structural economic constraints. The longitudinal comparison with an optimized synthetic counterfactual is consistent with the interpretation that the lack of policy evaluation has led to numerous interventions and analyses that have failed to provide effective solutions to depopulation.

In conclusion, the case of Aguaviva illustrates the complexity of reversing demographic inertia through isolated interventions. While the policy under study achieved notable short-term success in altering the demographic trend, the SCM evidence of convergence after 2016 reveals that these impacts were only temporary and were eventually erased as the initial momentum dissipated. The persistence of path-dependent mechanisms and the lack of deep institutionalization of change appear to have constrained its long-term impact. This study does not invalidate immigration-based repopulation strategies, but it does underscore the need for a longitudinal and structural perspective. These findings invite us to reconsider what success truly means for policies of this nature. Their value cannot be measured solely by the temporary influx of new residents; rather, it lies in their capacity, unrealized in this case, to set in motion a genuine and lasting departure from long-standing trajectories of demographic and economic decline. For such a transformation to take root, it is not sufficient to attract arrivals, what is needed is a deeper renewal of the social fabric and economic foundations of rural communities, one capable of sustaining momentum well beyond the life of the policy itself, and of preventing the quiet reassertion of the structural forces that have long driven depopulation.

To address this issue, a future research project is proposed, focused on the evaluation of these policies and the development of relevant indicators. Additionally, it would be highly valuable to replicate the Aguaviva analysis 10 years after 2020 in order to assess whether a new impact can be observed 30 years after the policy was first implemented.

## Data Availability

Publicly available datasets were analyzed in this study. This data can be found here: https://www.ine.es/. Further supporting data and detailed statistical tables are available as Supplementary Material in Data Sheet 1.
