# Supplementary material for: Depopulation, immigration, and gender dynamics: a case study of a long-term evaluation
Source: Front Sociol. 2026 Apr 30;11:1769829. doi: 10.3389/fsoc.2026.1769829 (PMC13171404; doi:10.3389/fsoc.2026.1769829)
Supplement: Supplementary file 1 [file Data_Sheet_1.zip › D. Methodological Appendix and Procedures.docx]

**Methodological Appendix**

# Introduction: Operationalizing the Institutional Framework

This methodological appendix details the analytical procedures employed to evaluate the long-term demographic impact of Aguaviva’s repopulation policy. The research design operationalizes the New Institutionalism in Sociology (NIS) framework by testing whether the municipal intervention disrupted path-dependent demographic trajectories characteristic of rural depopulation.

The Differences-in-Differences (DiD) model isolates the treatment effect, allowing us to empirically assess whether the policy generated a sustained deviation from structural decline or merely a temporary divergence followed by institutional convergence. This quasi-experimental approach, combined with robust validation procedures, provides causal inference within the constraints of observational longitudinal data.

1. Research Design and Data

## 1.1. Sample Construction and Selection Criteria

The original database comprised 731 municipalities across the autonomous community of Aragón (Spain). To ensure a homogeneous universe of comparable rural municipalities, the following inclusion criteria were applied:

Inclusion criteria: Population size: fewer than 2,000 inhabitants - Geographic location: outside the Zaragoza Metropolitan Area, following López Jiménez et al. (2002).

Rationale for exclusion criteria: The exclusion of municipalities within the Zaragoza Metropolitan Area was necessary to avoid the confounding influence of urban or peri-urban demographic dynamics, which operate under fundamentally different institutional and economic logics than rural depopulation processes.

Final analytical sample: After applying these criteria, the analytical sample consisted of 663 municipalities, representing 90.70% of Aragón’s territorial units and 19.39% of its population in the year 2000.

Group classification: The 663 municipalities were stratified into five analytical groups based on population size and policy intervention status:

Group 1: Aguaviva (treatment case, n=1).

Group 2: Members of the Spanish Association of Municipalities Against Depopulation (AEMD), excluding Aguaviva (n=13).

Group 3: Municipalities with 1,000–2,000 inhabitants, no known intervention (n=51).

Group 4: Municipalities with 500–999 inhabitants, no known intervention (n=84).

Group 5: Municipalities with fewer than 500 inhabitants, no known intervention (n=514).

This stratification allows for multiple levels of comparison: Aguaviva vs. municipalities with similar policy interventions (Group 2), and Aguaviva vs. size-matched control groups without intervention (Groups 3–5).

## 1.2. Data Sources and Temporal Scope

Primary data sources: Spanish National Statistics Institute (Instituto Nacional de Estadística, INE) and Aragonese Institute of Statistics (Instituto Aragonés de Estadística, IAEST).

Temporal scope: The analysis covers a 20-year period from 2000 to 2020, divided into two phases:

1. Pre-intervention period (2000–2008): Baseline phase preceding the full implementation and consolidation of Aguaviva’s repopulation policy.
2. Post-intervention period (2009–2020): Period following policy consolidation, marked by the 2008 global financial crisis and subsequent demographic adjustments.

Temporal resolution: Annual population data disaggregated by sex and nationality (Spanish vs. foreign residents).

Policy implementation timeline: While Aguaviva’s repopulation program formally began in 2000, the year 2009 was selected as the temporal cutoff for the DiD analysis for two substantive reasons:

1. Policy consolidation: By 2009, the initial wave of immigrant settlement (2000–2005) and subsequent stabilization period (2006–2008) had concluded, allowing sufficient time for demographic effects to manifest.
2. Exogenous shock: The 2008 global financial crisis represented a critical juncture that tested the resilience of the policy’s demographic gains, making 2009 an appropriate threshold to evaluate long-term sustainability.

## 1.3. Missing Data Treatment

Listwise deletion approach: For model estimation, a listwise deletion procedure (*complete case analysis*) was applied to cases presenting incomplete data on key grouping or temporal variables. This approach ensures:

1. Consistency in longitudinal estimation: Prevents distortions from incomplete temporal series.
2. Validity of interaction effects: Ensures the Group × Time interaction term in the DiD model is estimated on the same set of municipalities across both periods.

Justification: Given the high-quality administrative data from INE/IAEST, missingness was minimal (<2% of cases) and occurred randomly (Missing Completely at Random, MCAR), making listwise deletion appropriate without introducing systematic bias.

# 2. Analytical Strategy

## 2.1. Quasi-Experimental Design: Differences-in-Differences (DiD)

Methodological transition: The research evolved from a descriptive comparative framework to a causal impact evaluation model using Differences-in-Differences (DiD). This transition addresses the core theoretical question: Did Aguaviva’s institutional intervention alter its demographic trajectory relative to the counterfactual scenario (no intervention)?

DiD logic: The DiD estimator isolates the net effect of the policy by subtracting the natural trend observed in control municipalities from Aguaviva’s observed change. This “double differencing” removes:

1. Time-invariant group differences (e.g., Aguaviva’s baseline characteristics).
2. Common time trends (e.g., regional demographic decline).

What remains is the treatment effect attributable specifically to the policy intervention.

## 2.2. Standardization Procedure: Z-scores

Motivation for standardization: Given the heterogeneity in population size across municipalities (ranging from <100 to nearly 2,000 inhabitants), raw population counts are not directly comparable. A municipality losing 50 inhabitants represents: - 50% decline if initial population = 100 - 2.5% decline if initial population = 2,000.

Z-score transformation: To enable statistically valid comparisons, all demographic variables were transformed into standardized scores (Z-scores):

$$Z_{it}=\frac{X_{it}-\mu_{t}}{\sigma_{t}}$$

Where: - $X_{it}$ = raw value for municipality *i* at time *t* - $\mu_{t}$ = mean across all municipalities at time *t* - $\sigma_{t}$ = standard deviation across all municipalities at time *t.*

Properties of Z-scores: Mean = 0, Standard Deviation = 1 - Positive values indicate above-average performance; negative values indicate below-average performance - Magnitude indicates distance from the mean in standard deviation units.

Application: Z-scores were computed annually for: 1. Total population 2. Foreign population 3. Spanish population 4. Female population 5. Male population 6. Masculinity Index (males per 100 females).

This standardization allows the DiD model to detect *relative* deviations from regional trends, regardless of absolute population size.

## 2.3. Statistical Model Specification

General Linear Model (GLM) framework: The DiD estimator was implemented using IBM SPSS Statistics v.23 through General Linear Models (GLM) with a two-way ANOVA structure.

Model equation:

$$Y_{it}=\beta_{0}+\beta_{1}\cdot\text{Treat}_{i}+\beta_{2}\cdot\text{Post}_{t}+\beta_{3}\cdot\left( \text{Treat}_{i}\times\text{Post}_{t} \right)+\varepsilon_{it}$$

Where:

- $Y_{it}$ = Standardized demographic outcome (Z-score) for municipality *i* at time *t*
- $\text{Treat}_{i}$ = Binary treatment indicator:
  - 1 if municipality *i* = Aguaviva
  - 0 if municipality *i* ∈ control groups
- $\text{Post}_{t}$ = Binary time indicator:
  - 1 if year *t* ≥ 2009 (post-intervention period)
  - 0 if year *t* < 2009 (pre-intervention period)
- $\beta_{3}$ = DiD estimator (coefficient on the interaction term $\text{Treat}_{i}\times\text{Post}_{t}$)
- $\varepsilon_{it}$ = Error term

Interpretation of coefficients:

- $\beta_{0}$ = Baseline mean for control groups in pre-intervention period
- $\beta_{1}$ = Pre-intervention difference between Aguaviva and controls (should be ≈0 if parallel trends hold)
- $\beta_{2}$ = Common time trend affecting all municipalities
- $\beta_{3}$ **=** Treatment effect = Additional change in Aguaviva beyond the common trend

Key assumptions:

1. Parallel trends assumption: In the absence of treatment, Aguaviva would have followed the same trajectory as control municipalities.
2. No anticipation effects: Treatment effects begin only after policy implementation (post-2009).
3. Stable Unit Treatment Value Assumption (SUTVA): No spillover effects between municipalities (i.e., Aguaviva’s policy does not affect neighboring municipalities’ demographics).
4. Common shocks: Time-varying confounders (e.g., economic crises) affect treatment and control groups equally.

Significance testing: Statistical significance was assessed using F-tests for the DiD interaction term, with significance thresholds: - *** p < 0.01 (highly significant) - ** p < 0.05 (significant) - * p < 0.10 (marginally significant).

# 3. Robustness Checks and Validation

## 3.1. Placebo Test: Pre-Intervention Period (2000–2003)

Purpose: To validate the parallel trends assumption, a placebo test was conducted by simulating a “fake” intervention in the pre-treatment period.

Procedure: A DiD model was estimated for the period 2000–2003, treating the year 2001 as a hypothetical intervention point. Under the null hypothesis (parallel trends), there should be no significant “treatment effect” during this period when no actual intervention occurred.

Results: Interaction term (Group × Time): *p* = 0.812 (non-significant) - Interpretation: Parallel trends assumption validated.

Critical importance: This null result confirms that: 1. Aguaviva did not exhibit a divergent demographic trajectory *before* the policy implementation 2. Observed post-2009 differences are attributable to the policy, not to pre-existing structural differences or fortuitous trends 3. Selection bias and pre-treatment endogeneity are absent

## 3.2. Parallel Trends Assumption: Visual and Statistical Verification

Visual inspection: Graphical analysis of mean Z-scores for Aguaviva vs. control groups (2000–2008) showed overlapping trajectories, with no systematic divergence prior to intervention.

Statistical test: The non-significant placebo test (*p* = 0.812) provides formal evidence of parallel pre-treatment trends.

Robustness to violations: Even minor deviations from perfect parallel trends can be tolerated if: - Pre-treatment differences are time-invariant (absorbed by $\beta_{1}$) - Post-treatment divergence is substantially larger than any pre-existing gaps.

Both conditions are satisfied in this case.

## 3.3. Statistical Significance and Model Fit

Model diagnostics:

For the primary DiD models (total population and masculinity index), the following diagnostics were assessed:

1. F-statistic for overall model: Tests whether the model explains a significant portion of variance.
2. R² (Coefficient of Determination): Proportion of variance explained by the model.
3. *p*-values for individual coefficients: Significance of treatment effect, time effect, and interaction.

Results summary:

Model 1: Total Population (Z-scores) F-statistic: 18.45, *p* < 0.001 (model is significant) - R²: 0.366 (model explains 36.6% of variance) - DiD interaction term: *p* = 0.044** (significant at α = 0.05).

Model 2: Masculinity Index (Z-scores) F-statistic: 3.12, *p* = 0.030** (model is significant) - R²: 0.089 (model explains 8.9% of variance) - DiD interaction term: *p* = 0.047** (significant at α = 0.05).

Interpretation: Both models demonstrate statistically significant treatment effects, with the population model showing stronger explanatory power. The lower R² for the masculinity index reflects the greater volatility of sex ratios in small populations, but the significant interaction term confirms a robust gender-differentiated policy effect.

## 3.4. Robustness Analysis: Synthetic Control Method (SCM)

Although the DiD model provides a robust framework for group comparison, Aguaviva’s single-case nature raises concerns regarding internal validity. To mitigate potential selection bias and strengthen causal inference, the Synthetic Control Method (SCM) was incorporated. This approach constructs an optimized counterfactual through a weighted combination of comparable municipalities.

Estimation Procedure

Donor Pool: The control group consisted of 649 rural municipalities. This set was obtained by excluding from the total sample (663 municipalities) the treated unit (Aguaviva) and the 13 municipalities subject to similar repopulation policies (Group 2 – AEMD), thereby avoiding counterfactual contamination.

Pre-intervention Period: Optimization was conducted over the 1996–1999 period, excluding 1997 due to unavailable statistical data.

Predictor Variables and Joint Optimization: The synthetic unit was constructed using the historical trajectories of total, male, and female population. Unlike independent estimations by variable, a joint optimization was implemented imposing a single weight vector (*W*), selected through minimization of the pre-treatment Root Mean Squared Prediction Error (RMSPE). This strategy ensures structural demographic coherence (Total = Men + Women) and prevents internally inconsistent synthetic units.

Inference and Placebo Tests: Given the limitations of conventional significance testing in single-case studies, statistical validity was evaluated through in-space placebo tests, iteratively applying SCM to each of the 649 municipalities in the donor pool.

The empirical p-value based on the Post/Pre RMSPE ratio was 0.2126, placing Aguaviva in the upper segment of the placebo distribution under non-parametric inference.

# 4. Descriptive Statistics and Supplementary Analyses

## 4.1. Central Tendency and Dispersion Measures

Annual computation of descriptive statistics: For each analytical group and year, the following measures were calculated:

- Mean (μ): Central tendency
- Median: Robust central tendency measure (less sensitive to outliers)
- Standard Deviation (σ): Dispersion around the mean
- Minimum and Maximum: Range of values
- Skewness: Asymmetry of the distribution (to assess normality)

Purpose: Medians provide robustness checks against extreme values in small municipalities - Skewness tests whether Z-score distributions approximate normality - Minimum/maximum values identify outliers requiring further investigation.

## 4.2. Temporal Dynamics and Variation Rates

Inter-annual variation rates: Percentage change in population was computed for key sub-periods:

$$\text{Variation}_{t_{1}\to t_{2}}=\left( \frac{\text{Pop}_{t_{2}}-\text{Pop}_{t_{1}}}{\text{Pop}_{t_{1}}} \right)\times100$$

Key periods analyzed: 2000–2008 (pre-intervention) - 2009–2020 (post-intervention) - 2000–2020 (total period).

Slope analysis: Linear regression slopes (β coefficients) were estimated for each group to quantify the *rate of change* in population growth/decline over time. This allows comparison not only of final outcomes but also of the *velocity* of demographic processes.

## 4.3. Foreignization Ratio and Masculinity Index

Foreignization Ratio:

$$\text{Foreignization Ratio}=\frac{\text{Foreign Population}}{\text{Total Population}}\times100$$

This metric captures the relative weight of foreign residents and was critical for identifying: - Peak foreign population intensity (2012: 24.24% in Aguaviva) - Subsequent stabilization and decline.

Masculinity Index:

$$\text{Masculinity Index}=\frac{\text{Male Population}}{\text{Female Population}}\times100$$

This demographic indicator reveals gender imbalances and was central to detecting the unintended policy effect: greater female retention in Aguaviva relative to control groups.

Variance reduction analysis: The standard deviation of the foreignization ratio across municipalities decreased from 67.6 (2003) to 4.6 (2020), indicating *demographic convergence,* Aguaviva’s initial “positive anomaly” gradually homogenized with the rural environment.

# 5. Software and Replication Materials

Statistical software: Primary analyses and Differences-in-Differences (DiD) models were conducted using IBM SPSS Statistics v.23.

Replication materials (provided as supplementary files):

1. SPSS Syntax File (.sps): Complete command syntax for DiD models, including:

- Data transformations (Z-score computation).
- GLM specifications.
- Placebo test commands.
- Descriptive statistics generation.

1. SPSS Output File (.spv): Full output viewer file containing:

- Model estimation results.
- Diagnostic statistics.
- Descriptive tables.
- Graphical outputs.

1. **Data File (.xlsx):** Excel dataset containing all municipalities, classified by groups, with information on total population, population by gender, and population by origin (Spanish/foreign). This file allows for subsequent analyses using SPSS or other statistical software.

Reproducibility statement: All analytical procedures are fully documented in the syntax file, enabling exact replication of results. The syntax includes comments explaining each step, following best practices for transparent and reproducible research (Christensen et al., 2019).

Synthetic Control Method (SCM) Environment: Due to the computational intensity of the optimization algorithms and permutation tests, particularly the iterative processing of the 649 municipalities in the donor pool, the SCM analysis was conducted in a cloud-based computing environment using Google Colab and Python 3.x.

Implementation: Mathematical optimization libraries (such as scipy.optimize and cvxpy) were employed to estimate the weight vector W and to implement the leave-one-out procedures and cross-placebo tests across the full analytical sample.

Reproducibility: A Jupyter Notebook (.ipynb) is provided as supplementary material. This file contains the fully documented Python code, enabling exact replication of the synthetic counterfactual construction and the associated robustness tests.

# 6. Limitations and Scope

## 6.1. Internal Validity

Strengths: Parallel trends validated through placebo test - Homogeneous sample (rural municipalities < 2,000 inhabitants) - Long observation period (20 years).

Limitations: Quasi-experimental design (not randomized controlled trial) - Potential unmeasured confounders (e.g., local leadership quality, social capital) - Single treatment case (Aguaviva) limits statistical power for subgroup analyses.

## 6.2. External Validity

Generalizability: Results are directly generalizable to: Small rural municipalities in Mediterranean Europe with similar depopulation dynamics - Policy contexts combining immigrant attraction with minimal structural economic transformation.

Boundary conditions: Results may not generalize to: Larger towns or cities with diversified economies - Regions with sustained immigration flows independent of policy interventions - Contexts with fundamentally different institutional arrangements (e.g., Northern European welfare states).

## 6.3. Measurement Validity

Data quality: Administrative registry data from INE/IAEST are highly reliable, with minimal measurement error.

Construct validity: Population counts are direct measures (high construct validity) - “Repopulation policy success” is operationalized through population change, which may not capture qualitative dimensions (e.g., social cohesion, economic vitality).

# References

Angrist, J. D., & Pischke, J.-S. (2009). *Mostly Harmless Econometrics: An Empiricist’s Companion*. Princeton University Press.

Christensen, G., Freese, J., & Miguel, E. (2019). Transparent and Reproducible Social Science Research: How to Do Open Science. *California Management Review*, 61(2), 22–47.

DiMaggio, P. J., & Powell, W. W. (1983). The Iron Cage Revisited: Institutional Isomorphism and Collective Rationality in Organizational Fields. *American Sociological Review*, 48(2), 147–160.

López Jiménez, J. J., et al. (2002). *Delimitación del Área Metropolitana de Zaragoza*. Instituto Aragonés de Estadística.

Mahoney, J., & Thelen, K. (2010). *Explaining Institutional Change: Ambiguity, Agency, and Power*. Cambridge University Press.

Pierson, P. (2000). Increasing Returns, Path Dependence, and the Study of Politics. *American Political Science Review*, 94(2), 251–267.

Stockdale, A. (2006). Migration: Pre-requisite for rural economic regeneration? *Journal of Rural Studies*, 22(3), 354–366.
